# Sleep onset hypoventilation in chronic spinal cord injury

**DOI:** 10.14814/phy2.12490

**Published:** 2015-08-19

**Authors:** Amy T Bascom, Abdulghani Sankari, Harry G Goshgarian, M Safwan Badr

**Affiliations:** 1 Sleep Research Laboratory, John D. Dingell Veterans Affairs Medical CenterDetroit, MI, USA; 2 Wayne State University School of MedicineDetroit, MI, USA

**Keywords:** Apnea, hypoventilation, sleep, spinal cord injury, tetraplegia

## Abstract

A high prevalence of sleep-disordered breathing (SDB) after spinal cord injury (SCI) has been reported in the literature; however, the underlying mechanisms are not well understood. We sought to determine the effect of the withdrawal of the wakefulness drive to breathe on the degree of hypoventilation in SCI patients and able-bodied controls. We studied 18 subjects with chronic cervical and thoracic SCI (10 cervical, 8 thoracic SCI; 11 males; age 42.4 ± 17.1 years; body mass index 26.3 ± 4.8 kg/m^2^) and 17 matched able-bodied subjects. Subjects underwent polysomnography, which included quantitative measurement of ventilation, timing, and upper airway resistance (R_UA_) on a breath-by-breath basis during transitions from wake to stage N1 sleep. Compared to able-bodied controls, SCI subjects had a significantly greater reduction in tidal volume during the transition from wake to N1 sleep (from 0.51 ± 0.21 to 0.32 ± 0.10 L vs. 0.47 ± 0.13 to 0.43 ± 0.12 L; respectively, *P* < 0.05). Moreover, end-tidal CO_2_ and end-tidal O_2_ were significantly altered from wake to sleep in SCI (38.9 ± 2.7 mmHg vs. 40.6 ± 3.4 mmHg; 94.1 ± 7.1 mmHg vs. 91.2 ± 8.3 mmHg; respectively, *P* < 0.05), but not in able-bodied controls (39.5 ± 3.2 mmHg vs. 39.9 ± 3.2 mmHg; 99.4 ± 5.4 mmHg vs. 98.9 ± 6.1 mmHg; respectively, *P* = ns). R_UA_ was not significantly altered in either group. In conclusion, individuals with SCI experience hypoventilation at sleep onset, which cannot be explained by upper airway mechanics. Sleep onset hypoventilation may contribute to the development SDB in the SCI population.

## Introduction

Patients with spinal cord injury suffer from poor nocturnal sleep, sleep fragmentation, and high prevalence of sleep-disordered breathing (SDB) (Bonekat et al. 1990; Klefbeck et al. 1998; Burns et al. 2000; Berlowitz et al. 2005; Sankari et al. 2014a). However, the underlying mechanisms are not understood. What is clear, is that SDB in chronic SCI poses a significant quality of life issue for this population due to excessive daytime sleepiness, chronic fatigue, and cognitive impairment (Ayas et al. 2014; Sankari et al. 2014a; Vaessen et al. 2015), as well as increased risk of cardiovascular morbidity and mortality that accompanies underdiagnosed and untreated SDB (Caples et al. 2007; Marshall et al. 2014).

The prevalence of SDB after SCI has been reported as being between 27% and 77% (Bonekat et al. 1990; McEvoy et al. 1995; Klefbeck et al. 1998; Burns et al. 2000; Stockhammer et al. 2002; Berlowitz et al. 2005; Biering-Sorensen et al. 2009; Tran et al. 2010; Sankari et al. 2014a,b) as opposed to the prevalence of sleep apnea syndrome in the noninjured population, which is estimated to occur in approximately 2–4% (depending on age and gender) according to data from the Wisconsin Sleep Cohort Study (Peppard et al. 2013). Work from our laboratory has revealed that three out of four chronic SCI patients have symptomatic SDB, with central SDB noted in cervical SCI and obstructive SDB in thoracic SCI (Sankari et al. 2014a,b). Furthermore, we found that a narrowed CO_2_ reserve in patients with cervical SCI was associated with increased steady-state plant gain, which reflects the effectiveness of the respiratory system to eliminate CO_2_ for a given alveolar ventilation level. Accordingly, increased steady-state plant gain may promote the development of central apnea upon transition to non-REM sleep when breathing is mainly dependent on chemical stimuli. However, the etiology of increased plant gain and breathing instability during sleep in this SCI population, who may have normal gas exchange during wakefulness, versus able-bodied individuals is not known.

We hypothesized that individuals with SCI would develop a greater degree of sleep-related hypoventilation compared to able-bodied controls. To this end, we measured ventilation and upper airway resistance during transitions from alpha (wake) to theta (stage N1 sleep).

## Methods

### Subjects

Protocols were approved by the Human Investigation Committee of the John D. Dingell Veterans Affairs Medical Center and Wayne State University (Detroit, MI) and written informed consent was obtained.

We studied adults (≥18 years old) with chronic SCI and able-bodied participants if they met the inclusion and exclusion criteria. All subjects were instructed not to have alcohol, caffeine products, or sedatives on the day of the study.

#### Inclusion criteria

Participants with chronic SCI (>6 months postinjury), spanning the spectrum from cervical (cSCI, C4–C7) to thoracic levels (tSCI, T1–T6) (complete and incomplete injuries). Able-bodied subjects (AB) were recruited with similar demographics to the SCI group for age, body mass index (BMI), and gender.

#### Exclusion criteria

Participants were excluded from the study for any of the following: (1) pregnant or lactating females; (2) currently ventilator dependent or with tracheostomy tube in place; (3) history of cardiac disease including heart failure, peripheral vascular disease, or stroke; (4) history of head trauma resulting in neurological symptoms or loss of consciousness; (5) advanced lung, liver, or chronic kidney disease; (6) extreme obesity, defined for this protocol as BMI > 38 kg/m^2^; or (7) other illness that would interfere with completion of the study.

The first visit to the laboratory consisted of medical history, physical examination that included vital signs, maximal inspiratory and expiratory pressures (MIP and MEP) for SCI individuals, and spirometry to rule out pulmonary disease. The second visit consisted of an overnight polysomnography (PSG). If the subject had a concern about sleep difficulties, Zolpidem was administered orally 30 min prior to beginning of recordings. Zolpidem dose was selected based on the subject’s age (≥60 years old: 5 mg, <60 years: 10 mg IR or 12.5 mg CR). The number of subjects requiring Zolpidem was similar in both groups (Table1).

**Table 1:** Patient characteristics

	SCI	AB	*P*
*N*	18	17	ns
Age (years)	42.4 ± 17.1	42.9 ± 13.9	ns
BMI (kg/m^2^)	26.3 ± 4.8	27.8 ± 5.4	ns
Gender (M/F)	11/7	8/9	ns
NC (cm)	38.5 ± 3.0	37.1 ± 3.7	ns
AHI (events/h)	22.9 ± 22.4	7.3 ± 8.8	<0.05
AHI (>5 events/h)	12	8	–
Zolpidem (Y/N)	9/9	8/7	ns
Injury level (cervical/thoracic)	10/8	–	–
MIP (% predicted)	87.2 ± 29.4	–	–
MEP (% predicted)	42.3 ± 15.8	–	–
FVC (% predicted)	71.5 ± 17.0	86.5 ± 26.5	NS
FEV 1 (% predicted)	76.4 ± 16.7	87.6 ± 18.4	NS
FEV1/FVC	80.5 ± 7.0	77.6 ± 9.6	NS

All data mean ± SD. SCI, spinal cord injury; AB, able-bodied; BMI, body mass index; NC, neck circumference; AHI, apnea hypopnea index; MIP, maximal inspiratory pressure; MEP, maximal expiratory pressure; FVC, forced vital capacity; FEV1, forced expiratory volume at 1 sec; FEV1/FVC, the ratio of FEV1 to FVC; NS, not significant.

### Polysomnography

Subjects arrived at the laboratory between 8:00 and 9:00 pm to be instrumented and prepared for study. PSG was performed in the supine position using the Comet PSG System (AS40 Model) or the Heritage II PSG System (Grass Technologies, Warwick, RI). Measurements included electrocardiogram, electroencephalogram (EEG), electro-oculograms (EOG) and chin electromyogram using the International 10–20 system of electrode placement (EEG: C3-A2 and C4-A2; EOG: O-A2). Subjects wore a nasal mask (Respironics Profile Lite, Respironics Inc., Murrysville, PA) connected to a pneumotachometer (Hans Rudolph, Model 3700A, Shawnee, KS) that measured airflow. The mask was adjusted to minimize leaks, and glued in place to reduce leaks when required. Tidal volume (*V*_T_) was determined via integration of the pneumotachometer flow signal. End-tidal carbon dioxide (P_E__T_co_2_) and end-tidal oxygen (P_E__T_o_2_) levels were measured with CO_2_ and O_2_ gas analyzers (Vacumed Model 17515 and 17518 respectively, Ventura, CA). Supraglottic airway pressure was measured with a pressure tipped catheter (Millar Instruments, Houston, TX) placed through one nostril and extending down into the hypopharynx at least 2 cm caudal to the visible base of the tongue and superior to the epiglottis. Pulse oximetry was measured via ear probe (Biox 3740; Datex-Ohmeda Inc., Madison, WI). Subjects were recorded while breathing spontaneously on room air. Ventilation data from the pneumotachometer, supraglottic catheter, pulse oximeter, and gas analyzers were digitized and analyzed using a PowerLab Data Acquisition System (Model 16SP, ADInstruments Inc., Colorado Springs, CO).

### Data analysis

Polysomnographys were scored using American Academy of Sleep Medicine (AASM) 2012 recommended criteria (Berry et al. 2012). Supraglottic pressure and respiratory inductance plethesmography bands (Respitrace, model 200; Nims Inc., Miami Beach, FL) were used to differentiate between obstructive and central apneas. In order to analyze state-specific changes in ventilation, transitions between wake and sleep were identified first, with sleep stage scorers blinded to ventilation. Blinding of ventilation signals was accomplished by covering the portion of the screen that contained ventilation signals. Two independent sleep scorers identified and verified agreement of wake to sleep transitions. Occipital EEG signals were used to determine the predominance of alpha (resting wakefulness with eyes closed, 8–12 Hz) versus theta (stage N1 sleep, 4–7 Hz) waves without K complexes or sleep spindles.

**Figure 1:**
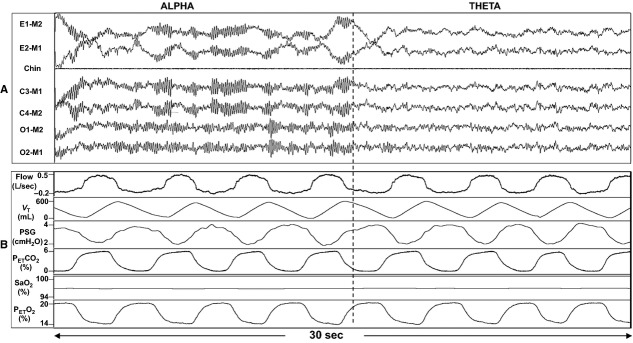
(A) A 30-sec representative polygraph with electroencephalogram (EEG) and electromyogram (EMG) recordings in a 55-year-old able-bodied subject (male, body mass index 27 kg/m^2^) during the transition from wake (alpha) to N1 sleep (theta). E, eye, M, mastoid ground; C, central; O, occipital. (B) A 30-sec polygraph of ventilation, time matched to the EEG data in panel A. *V*_T_, tidal volume; PSG, supraglottic pressure; P_E__T_co_2_, end-tidal CO_2_; P_E__T_o_2_, end-tidal O_2_.

After identification of three separate alpha to theta transitions, ventilation data were obtained and analyzed from time-matched segments as depicted in Figure1. In the case that three transition segments could not be identified for the subject, two transitions were used. Ventilation data obtained consisted of breath-by-breath minute ventilation (*V*_E_), tidal volume (*V*_T_), respiratory frequency (ƒ), inspiratory time (*T*_i_), expiratory time (*T*_e_), total cycle time (*T*_tot_), oxygen saturation (SaO_2_), P_E__T_co_2_ and P_E__T_o_2_. Inspiratory R_UA_ was calculated at the linear portion of the pressure–flow relationship using the pressure–flow loops (supraglottic pressure and airflow).

Alpha to theta transitions were only analyzed if there were at least two 30-sec epochs of wake preceding the transition so that brief arousals from sleep with corresponding hyperpneas were not used. We did not analyze any transitions from theta to alpha (arousals from sleep). Theta segments were not required to last beyond three breaths for analysis purposes, as arousal often occurred if theta breaths consisted of apnea or hypopneas. For each subject, all ventilatory parameters for alpha breaths were grouped and averaged as well as theta breaths and used for comparison between conditions and groups.

To verify the accuracy of visual scoring of segments selected for analysis, we performed spectral analysis using Fast Fourier Transform method (MATLAB, Math Works Inc., Natick, MA) on EEG segments selected for alpha and theta analysis in four subjects. We found >90% agreement between visual scoring and spectral analysis and thus proceeded with visual classification (Trinder et al. 1992; Yang et al. 2012).

In theta breaths, where significant hypopnea or apnea occurred, P_E__T_co_2_ signals were invalid in some breaths due to insufficient flow and lack of end-tidal plateau. In these cases, the P_E__T_co_2_ values were eliminated for these breaths. Thus, only breaths with reliable signals were used for P_E__T_co_2_ analysis. When apnea occurred, the total time of the apnea was considered as part of the Te of the breath before apnea, thus V_E_ for that breath (the breath before apnea) reflected the overall decrease in ventilation as a result of the apnea. Breaths where hypopnea occurred were analyzed in the same manner as all other breaths used for analysis.

Three secondary analyses were performed: (1) To determine if spinal injury level influenced sleep onset ventilation, sleep onset changes were compared between participants with cervical and thoracic SCI. (2) To determine the potential confounding effect of SDB on sleep onset ventilatory changes, we compared sleep onset changes in ventilation in eight cervical SCI individuals and eight able-bodied controls with SDB. (3) To determine the potential contribution of intercostal muscle atonia on sleep onset changes in ventilation, we analyzed the transition from non-REM to REM sleep in a subset of subjects in each of the following groups who had REM sleep: cervical SCI (*n* = 3), thoracic SCI (*n* = 2), and able-bodied subjects (*n* = 3). Data are reported as the mean ± SD.

### Statistical analysis

Two-way repeated measures ANOVA (Sigma Plot 12.1, Systat Software, Inc., San Jose, CA) was performed to determine within-group (e.g., alpha to theta changes in cSCI) and between-group differences in ventilatory parameters and upper airway resistance between the two conditions: alpha and theta EEG frequencies. When appropriate, post hoc pairwise multiple comparisons were made using the Student–Newman–Keuls method. When data were not normally distributed, appropriate nonparametric analysis was employed. *T*-tests were used to compare all demographic data between AB and SCI. All data are reported as mean ± SD and significance was set at *P *<* *0.05.

## Results

### Sleep onset ventilatory changes

**Figure 2:**
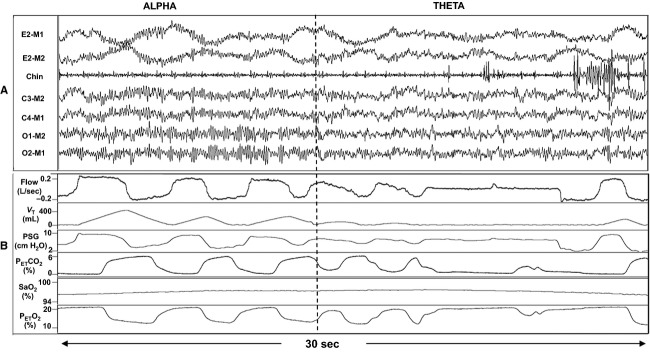
(A) A 30-sec representative polygraph with electroencephalogram (EEG) and electromyogram (EMG) recordings in a 36-year-old chronic cervical (C6, incomplete injury) spinal cord injury individual (male, body mass index 28.2 kg/m^2^) during the transition from wake (alpha) to N1 sleep (theta). E, eye; M, mastoid ground; C, central; O, occipital. (B) A 30-sec polygraph with ventilation recording, time matched to the EEG data in panel A. Note the reduction in flow and tidal volume with sleep onset. *V*_T_, tidal volume; PSG, supraglottic pressure; P_E__T_co_2_, end-tidal CO_2_; P_E__T_o_2_, end-tidal O_2_.

**Figure 3:**
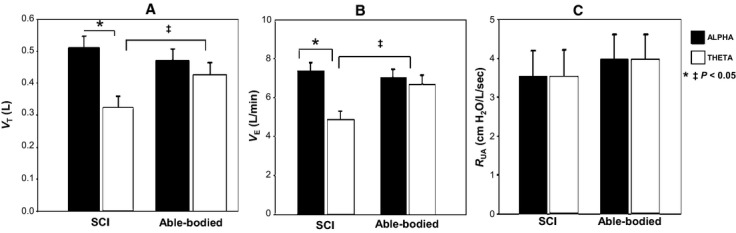
(A) Greater decrease in tidal volume (*V*_T_) with sleep onset in spinal cord injury (SCI) compared to able-bodied subjects. (B) Greater decrease minute ventilation (*V*_E_) at sleep onset in SCI group. (C) No significant change in upper airway resistance at sleep onset in either group. Bars are SEM.

We studied 18 subjects with SCI and 17 AB with similar demographics (Table1). In the SCI group, we analyzed 47 transitions (137 and 134 breaths in alpha and theta, respectively). In the AB group, we analyzed 40 transitions (112 and 108 breaths in alpha and theta, respectively). Figure1 illustrates the ventilatory changes in a representative AB subject. Figure2 illustrates the tidal volume (*V*_T_) decrease during the transition from alpha to theta in a representative cSCI subject. Group changes in *V*_T_ and *V*_E_ during sleep onset transitions are detailed in Figure3A and B. Sleep onset was associated with significant decrease in *V*_T_ and *V*_E_ in the SCI but not the AB subjects. However, there was no significant change in R_UA_ with sleep onset in either group (Fig.3C).

Table2 summarizes the effect of sleep onset on ventilatory parameters in SCI and AB groups. Sleep onset in the SCI group was associated with decreased *T*_i_ while lengthening *T*_e_, reduced duty cycle (*T*_i_/*T*_tot_) and unchanged respiratory frequency and *T*_tot_. In contrast, the AB group demonstrated increased respiratory frequency at sleep onset with no significant changes in *T*_i_, *T*_e_, *T*_tot_, or the duty cycle.

**Table 2:** Effect of sleep onset on respiratory cycle timing and chemical stimuli

	SCI		AB	
	Alpha	Theta	Alpha	Theta
Frequency (breaths/min)	15.3 ± 3.1	14.9 ± 3.9	15.3 ± 2.5	16.2 ± 2.9†
*T*_i_ (sec)	1.8 ± 0.4	1.7 ± 0.3†	1.8 ± 0.3	1.7 ± 0.4
*T*_e_ (sec)	2.4 ± 0.7	3.0 ± 1.6†	2.3 ± 0.5	2.2 ± 0.5*
*T*_i_/*T*_tot_	0.44 ± 0.05	0.40 ± 0.08†	0.43 ± 0.05	0.44 ± 0.07
SaO_2_ (%)	96.1 ± 1.4	95.8 ± 1.7	96.2 ± 1.0	96.2 ± 0.9
P_E__T_co_2_ (mmHg)	38.9 ± 2.7	40.6 ± 3.4†	39.5 ± 3.2	39.9 ± 3.2
P_E__T_o_2_ (mmHg)	94.1 ± 7.1	91.2 ± 8.3†	99.4 ± 5.4	98.9 ± 6.1

All data mean ± SD. SCI *n* = 18, AB *n* = 17. SCI, spinal cord injury; AB, able-bodied; *T*_i_, inspiratory time; *T*_e_, expiratory time, *T*_i_/*T*_tot_, ratio of Ti to total cycle time.

* Between-group (SCI vs. able-bodied) difference *P *<* *0.05.

† Within-group difference (alpha vs. theta) *P* < 0.05.

### Comparison of cervical versus thoracic SCI

To determine if spinal injury level influenced sleep onset ventilation, we compared the ventilatory parameters detailed above between cSCI (*n* = 10) and tSCI (*n* = 8) subjects. Alpha to theta transitions were associated with significantly decreased *V*_T_ in cSCI subjects (alpha: 576.0 ± 256.3 mL, theta: 293.1 ± 105.8 mL, *P* < 0.05) but not in tSCI (alpha: 445.2 ± 100.9 mL, theta: 364.6 ± 90.7 mL, *P* = ns). Similarly, *V*_E_ was significantly reduced in cSCI (alpha: 7.8 ± 2.6 L, theta: 4.3 ± 1.6 L, *P* < 0.05) compared to tSCI (alpha: 6.9 ± 1.5 L, theta: 5.6 ± 1.4 L, *P* = 0.08). Thus, injury level has a significant impact on sleep onset hypoventilation. Further results of cSCI versus tSCI analyses are detailed in Table3.

**Table 3:** Cervical versus thoracic SCI: sleep onset ventilation

	cSCI		tSCI	
	Alpha	Theta	Alpha	Theta
Frequency (breaths/min)	14.7 ± 3.3	14.2 ± 4.5	15.9 ± 3.0	15.7 ± 2.9
*T*_i_ (sec)	1.9 ± 0.4	1.7 ± 0.4†	1.7 ± 0.3	1.6 ± 0.3
*T*_e_ (sec)	2.5 ± 0.7	3.51 ± 2.0	2.3 ± 0.7	2.4 ± 0.6
*T*_i_/*T*_tot_	0.44 ± 0.04	0.38 ± 0.09†	0.43 ± 0.06	0.42 ± 0.06
SaO_2_ (%)	95.8 ± 1.3	95.1 ± 1.6	96.6 ± 1.4	96.7 ± 1.4*
P_E__T_co_2_ (mmHg)	38.9 ± 3.2	41.3 ± 4.1†	38.9 ± 1.7	39.5 ± 1.8
P_E__T_o_2_ (mmHg)	94.3 ± 4.8	90.6 ± 6.8†	93.8 ± 10.1	92.0 ± 10.6
R_UA_ (cmH_2_O/L/sec)	3.61 ± 1.7	3.5 ± 1.9	3.5 ± 1.4	3.6 ± 1.7

All data mean ± SD. cSCI *n* = 10, tSCI *n* = 8. cSCI, cervical SCI; tSCI, thoracic SCI. *T*_i_, inspiratory time; *T*_e_, expiratory time; *T*_i_/*T*_tot_, ratio of *T*_i_ to total cycle time; R_UA_, upper airway resistance.

* Between-group (cervical vs. thoracic) difference *P* < 0.05.

† Within-group difference (alpha vs. theta) *P* < 0.05.

### Effect of sleep disordered breathing on sleep onset hypoventilation in cSCI and AB subjects

To determine the potential contribution of SDB to sleep onset hypoventilation in the cSCI group; we compared eight cSCI individuals and eight AB subjects with SDB as defined by an AHI ≥ 5 events/h. Sleep onset was associated with decreased *V*_T_ and *V*_E_ in participants with cSCI and SDB, but not in AB subjects with SDB (Table4). Changes in other ventilatory parameters are detailed in Table4. Thus, cSCI with SDB have a significantly greater reduction in ventilation with sleep onset compared to AB subjects with SDB.

**Table 4:** Cervical SCI versus able-bodied subjects: effect of sleep disordered breathing on sleep onset ventilation

	cSCI		AB	
	Alpha	Theta	Alpha	Theta
Frequency (breaths/min)	14.4 ± 3.5	13.9 ± 5.1	15.7 ± 2.3	17.3 ± 3.2
*V*_E_ (L/min)	7.4 ± 2.6	4.4 ± 1.8†	6.6 ± 2.4	6.1 ± 2.1
*V*_T_ (L)	0.58 ± .26	0.30 ± 0.12†	0.43 ± 0.18	0.37 ± 0.16
*T*_i_ (sec)	2.0 ± 0.5	1.8 ± 0.4†	1.7 ± 0.1	1.5 ± 0.2
*T*_e_ (sec)	2.6 ± 0.7	3.8 ± 2.1†	2.2 ± 0.6	2.1 ± 0.7*
*T*_i_/*T*_tot_	0.44 ± 0.04	0.37 ± 0.09†	0.43 ± 0.06	0.44 ± 0.07
SaO_2_ (%)	95.8 ± 1.4	95.1 ± 1.8	96.0 ± 1.3	96.1 ± 1.1
P_E__T_co_2_ (mmHg)	39.3 ± 3.1	41.6 ± 3.4†	39.6 ± 2.6	39.8 ± 2.2
P_E__T_o_2_ (mmHg)	94.6 ± 5.0	91.4 ± 6.5†	101.1 ± 4.2	100.4 ± 5.9*
R_UA_ (cmH_2_O/L/sec)	3.4 ± 1.3	3.2 ± 1.7	4.3 ± 2.9	4.8 ± 5.2

All data mean ± SD. cSCI *n* = 8, AB *n* = 8. cSCI, cervical SCI; AB, able-bodied; *V*_E_, minute ventilation; *V*_T_, tidal volume; *T*_i_, inspiratory time; *T*_e_, expiratory time; *T*_i_/*T*_tot_, ratio of *T*_i_ to total cycle time; R_UA_, upper airway resistance.

* Between-group (cSCI vs. AB) difference *P* < 0.05.

† Within-group difference (alpha vs. theta) *P* < 0.05.

### Comparison of ventilation with REM onset in cervical and thoracic SCI and able-bodied subjects

To ascertain the relative contribution of the loss of intercostal muscle activity on sleep onset hypoventilation, we compared the ventilatory changes during non-REM to REM transitions. We reasoned that the magnitude of non-REM to REM ventilatory changes would be attenuated in individuals with cSCI because of the loss of intercostal muscle activity. There was a paucity of REM sleep in all subjects, owing to the high level of instrumentation in our protocols. We identified non-REM to REM transitions in a subset of three cSCI individuals, two tSCI subjects, and three AB subjects.

Figures S1, S2 can be found in the Supporting Information, and depict representative polygraphs of EEG frequencies and respiration during the transition from non-REM to REM sleep in a cSCI and AB subject, respectively. Able-bodied subjects had a larger drop in ventilation with REM onset than SCI subjects (REM *V*_E_ as % of NREM *V*_E_: cSCI: 103.7 ± 16.4%, tSCI: 82.5 ± 11.9%, AB: 74.3 ± 26.6%). See Table S1 in Supporting Information.

## Discussion

### Summary of findings

The purpose of this study was to identify the ventilatory changes at sleep onset during the transition from alpha to theta EEG frequencies. The major findings of our study were (1) sleep onset was associated with hypoventilation in individuals with chronic SCI compared to able-bodied subjects; (2) sleep onset hypoventilation was predominantly present in participants with cervical but not thoracic SCI; and (3) there was no change in upper airway resistance in either group.

### The effect of sleep onset on ventilation and timing in spinal cord injury

Our study demonstrated that the loss of the wakefulness stimulus to breathe was associated with decreased *V*_E_ and increased P_E__T_co_2_ in individuals with SCI but not in able-bodied participants. Decreased ventilation was not due to increased upper airway resistance. Thus, altered upper airway mechanics were not responsible for the sleep onset hypoventilation in individuals with SCI.

### Methodological considerations

Evidence in the literature suggests that sleep onset is associated with transient breathing instability and a decrease in ventilation, even in healthy individuals (Douglas et al. 1982b; Colrain et al. 1987; Trinder et al. 1992; Dunai et al. 1999). Trinder et al. (1992) reported a 19% drop in *V*_T_ during alpha to theta transitions in healthy adult males and a 13% decrease in females. We found no significant (<10%) decrease in *V*_T_ in theta compared with alpha in the AB group. One of the explanations for these differences in findings could be related to the fact that our study was carried out in one night with spontaneous transitions between sleep and wake in contrast to other studies which used induced arousals from sleep repeatedly in different nights (Trinder et al. 1992; Kay et al. 1996). Furthermore, Trinder et al. used healthy, young (average 20.4 years) subjects, while our study included wide range of ages with and without SDB. The difference in the findings between the two studies may be accounted for by the variations in the protocols, demographics of subjects utilized, and number of transitions and breaths considered for analysis.

**Figure 4:**
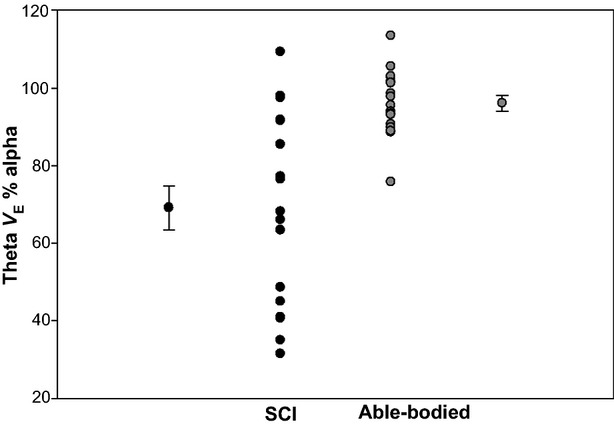
An illustration of the intersubject variability in the change in minute ventilation (*V*_E_) from alpha to theta electroencephalogram activity during sleep onset. spinal cord injury *n* = 18, able-bodied subject *n* = 17. Data points beside individual subject group data are the group means. Bars are SEM.

The amount of variability between subjects in regard to change in ventilation and frequency during sleep onset may be a factor in the lack of a significant reduction in *V*_E_ in able-bodied subject. Figures4, S3 and S4 depict the between subject variability for change in *V*_E_, V_T_, and frequency from alpha to theta.

Demographic parameter matching for BMI was done between SCI and able-bodied subjects. It is worth noting that BMI matching may not be accurately accomplished between able-bodied and SCI subjects because of loss of muscle mass in the SCI group. Therefore, SCI subject may have a higher percentage of body fat compared to their matched able-bodied counterparts.

In our subanalysis of able-bodied versus SCI subjects with SDB, the severity of SDB in the SCI group was greater than in the able-bodied group (33.5 ± 20.2 vs. 14.4 ± 8.2, respectively, *P* = 0.01). Therefore, it is possible that the severity of the SDB in SCI subjects makes the comparison difficult. We did not recruit our subjects on the basis of the presence of or severity of SDB. These findings were obtained after scoring the PSGs. We found that the SCI group had a greater occurrence and severity of SDB, which made close matching of AHI impossible with our number of subjects. A future study using a larger group of subjects with a similar severity of SDB would clarify this point.

### Putative mechanisms of sleep onset hypoventilation in SCI

The major finding in our study was a greater drop in *V*_T_ at sleep onset in SCI compared to able-bodied individuals. Moreover, *V*_T_ drop was related to the level of injury and was greater in cervical SCI, who have significant ventilatory instability in comparison to able-bodied subjects (Sankari et al. 2014a). Consistent with previous work (Kay et al. 1996), we found that upper airway resistance does not explain the decrease in ventilation at sleep onset. Therefore, other factors that may contribute to ventilatory instability during sleep onset include state-related fluctuations in the drive to the primary respiratory muscles and variability in compensatory mechanisms.

We considered several possible mechanisms of sleep onset hypoventilation in SCI subjects. First, chest wall deformities, such as in mid- and high thoracic SCI, might contribute to increased mechanical loading and reduced lung volume leading to hypoventilation (Castriotta and Murthy 2009). Second, reduced central ventilatory drive may cause hypoventilation in subjects with SCI, especially cervical SCI. There is evidence from animal studies that central ventilatory drive may be diminished in cervical SCI (Zimmer and Goshgarian 2007). This is unlikely in our study as there was no difference in ventilation or P_E__T_co_2_ during wakefulness in SCI versus able-bodied control subjects. Whether SCI is associated with a more pronounced sleep-related decrease in central ventilatory motor output cannot be determined from our data.

Third, sleep onset hypoventilation may be influenced by peripheral chemoreceptor activity. A previous study by Dunai et al. (1999) suggested an interaction between chemical stimuli and state effects on ventilation during sleep onset. Specifically, individuals with increased peripheral chemoreceptor activity displayed amplified state-related changes in ventilation and subsequent dampening following hyperoxic exposure. However, sleep onset hypoventilation was more pronounced in cervical SCI subjects even when compared to able-bodied individuals with SDB, a group that is known to have augmented peripheral chemoreceptor activity.

Finally, individuals with spinal cord injury are more susceptible to sleep onset hypoventilation than healthy individuals, owing to the denervation of some, or all, intercostal muscles (Terson de Paleville et al. 2011; Burns et al. 2000; Lyall et al. 2001). Evidence in the literature suggests that rib cage contribution to *V*_T_ increases by 20% during non-REM sleep relative to wakefulness (Tabachnik et al. 1981). Therefore, we interpret accentuated sleep onset hypoventilation in individuals with tetraplegia as secondary to loss of intercostal muscle activity. The attenuation of non-REM to REM hypoventilation in subjects with cervical spine injury supports this interpretation. However, our study precludes drawing firm conclusions regarding the relative contribution of the loss of intercostal muscle activity versus increased peripheral chemoreceptor activity on sleep onset hypoventilation. In summary, our study demonstrated an augmented sleep-related hypoventilation in patients with SCI, mostly in subjects with cervical SCI and without significant changes in upper airway resistance.

### Physiologic implications

Sleep onset hypoventilation may contribute to sleep-related breathing instability. Individuals with SCI can maintain stable sleep and alveolar ventilation despite modest hypoventilation. However, significant hypoventilation may occur in individuals with a high spinal level of SCI and abnormal respiratory mechanics or those who use CNS suppressing medications. We have previously shown that tetraplegia is a risk factor for central apnea owing to increased plant gain (Sankari et al. 2014b). Therefore, sleep onset hypoventilation may increase the propensity to develop central apnea by increasing plant gain. The ensuing sleep fragmentation may lead to ventilatory overshoot and recurrent episodes of apnea/hypopnea alternating with hyperpnea (Douglas et al. 1982a; Douglas 1992; Badr 2005; White 2005; Eckert et al. 2007; Yumino 2008; Nemati et al. 2011). Therefore, sleep onset hypoventilation may promote the development of recurrent central apnea and sleep-related breathing instability in subjects with SCI.

## Conclusions

We have shown the occurrence of significant sleep onset hypoventilation in patients with chronic SCI compared to able-bodied subjects. The magnitude of sleep onset hypoventilation is not associated with increased upper airway resistance and is related to the level of SCI. Diminished neuromuscular output owing to intercostal muscle paralysis could play a role in the development of sleep apnea postinjury to the cervical spine.

## Supporting Information

**Data S1.** Non-REM to REM sleep transition analysis (Iber et al. 2007).

**Figure S1.** NREM to REM transition in cervical SCI subject.

**Figure S2.** NREM to REM transition in able-bodied subject.

**Figure S3.** An illustration of the inter-subject variability in the change in tidal volume (VT) from Alpha to Theta EEG activity during sleep onset. SCI *n* = 18, Able-Bodied subject *n* = 17. Data points beside individual subject group data are the group means. Bars are SEM.

**Figure S4.** An illustration of the inter-subject variability in the change in respiratory frequency from Alpha to Theta EEG activity during sleep onset. SCI *n* = 18, Able-Bodied subject *n* = 17. Data points beside individual subject group data are the group means. Bars are SEM.

**Table S1.** Tidal volume during non-REM to REM transitions.
