# Supplementary material for: Sleep onset hypoventilation in chronic spinal cord injury
Source: Physiol Rep. 2015 Aug 19;3(8):e12490. doi: 10.14814/phy2.12490 (PMC4562576; doi:10.14814/phy2.12490)
Supplement: Supplementary file 1 [file phy20003-e12490-sd1.docx]

Supplementary Material

*Non-REM to REM sleep transition analysis*

One transition from non-REM to REM sleep was analyzed for each subject (3 transitions in cSCI and AB, 2 transitions in tSCI group). The 10 non-REM breaths immediately prior to REM onset were analyzed for tidal volume in each group, as well as for the first 10 breaths of REM sleep. Any breaths associated with an arousal were not included in the analysis. The onset of REM was determined using the AASM 2007 guidelines (17). REM segments exhibited the following features: low-amplitude, mixed-frequency EEG activity, absence of K complexes and sleep spindles and decrease in chin tone with rapid eye movements following within 1 epoch as determinants of the exact point of REM onset.
