# Supplementary figures and images for: Sleep onset hypoventilation in chronic spinal cord injury
Source: Physiol Rep. 2015 Aug 19;3(8):e12490. doi: 10.14814/phy2.12490 (PMC4562576; doi:10.14814/phy2.12490)

NREM

REM

A.

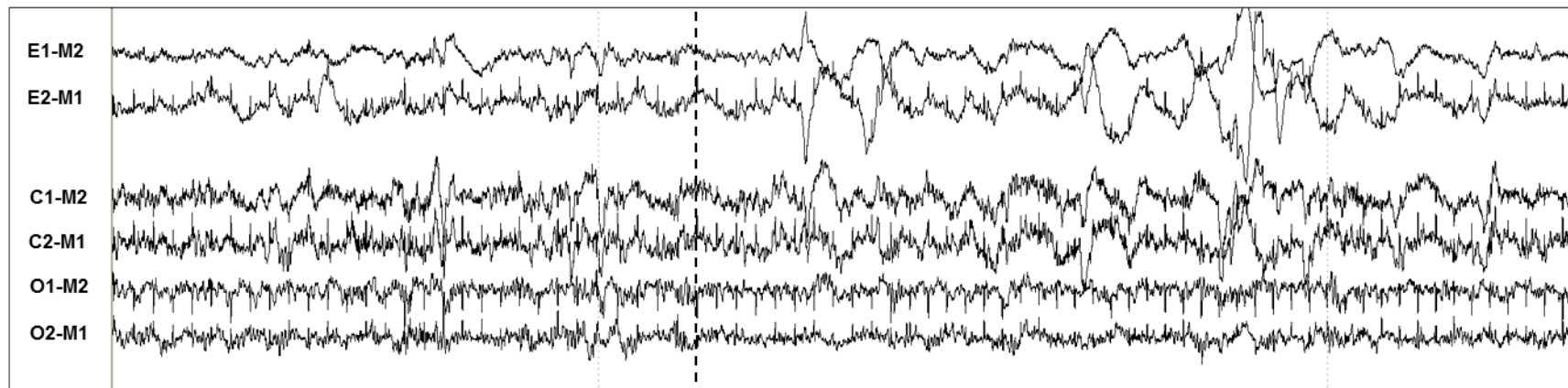

B.

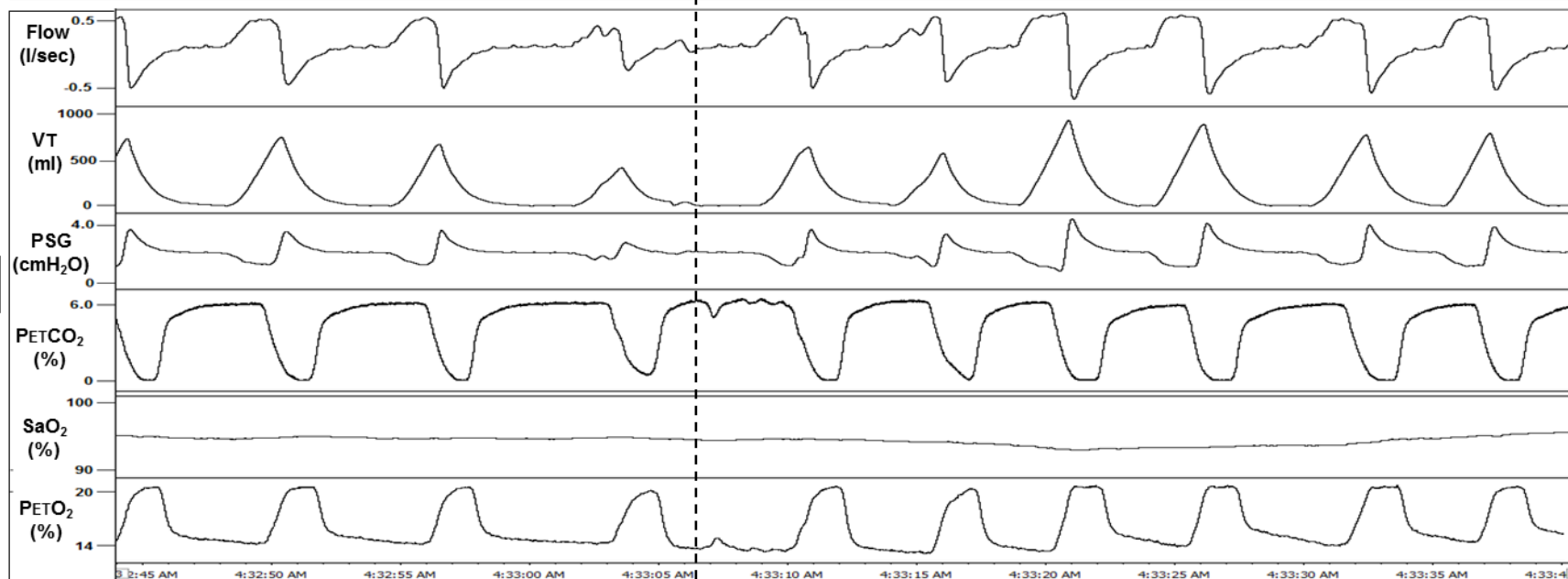

60 seconds

Supplement: Supplementary file 2 [file phy20003-e12490-sd2.pdf]

A.

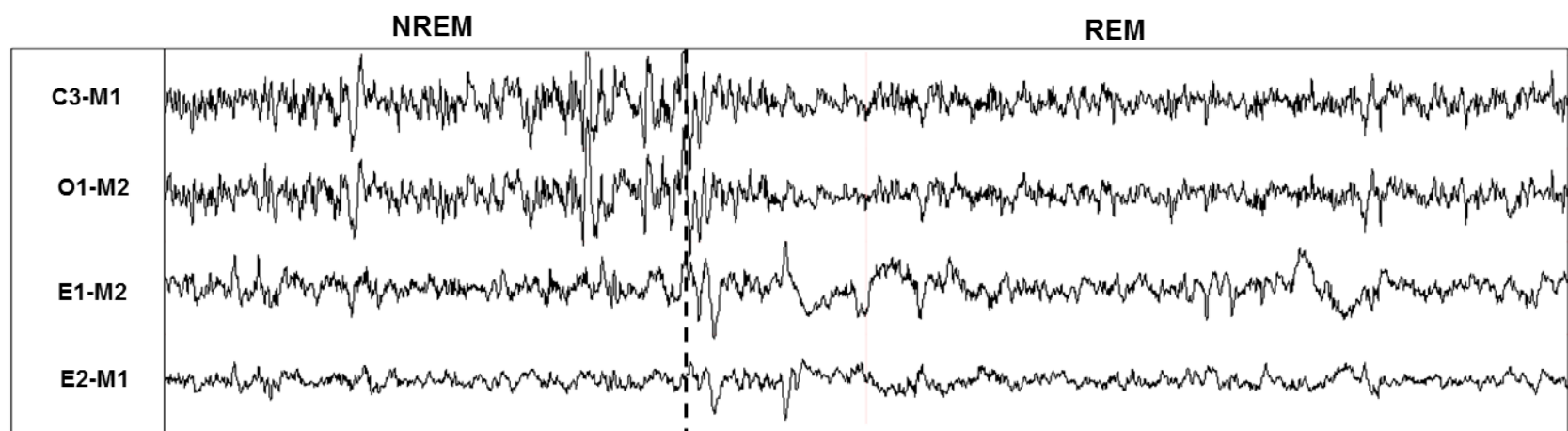

B.

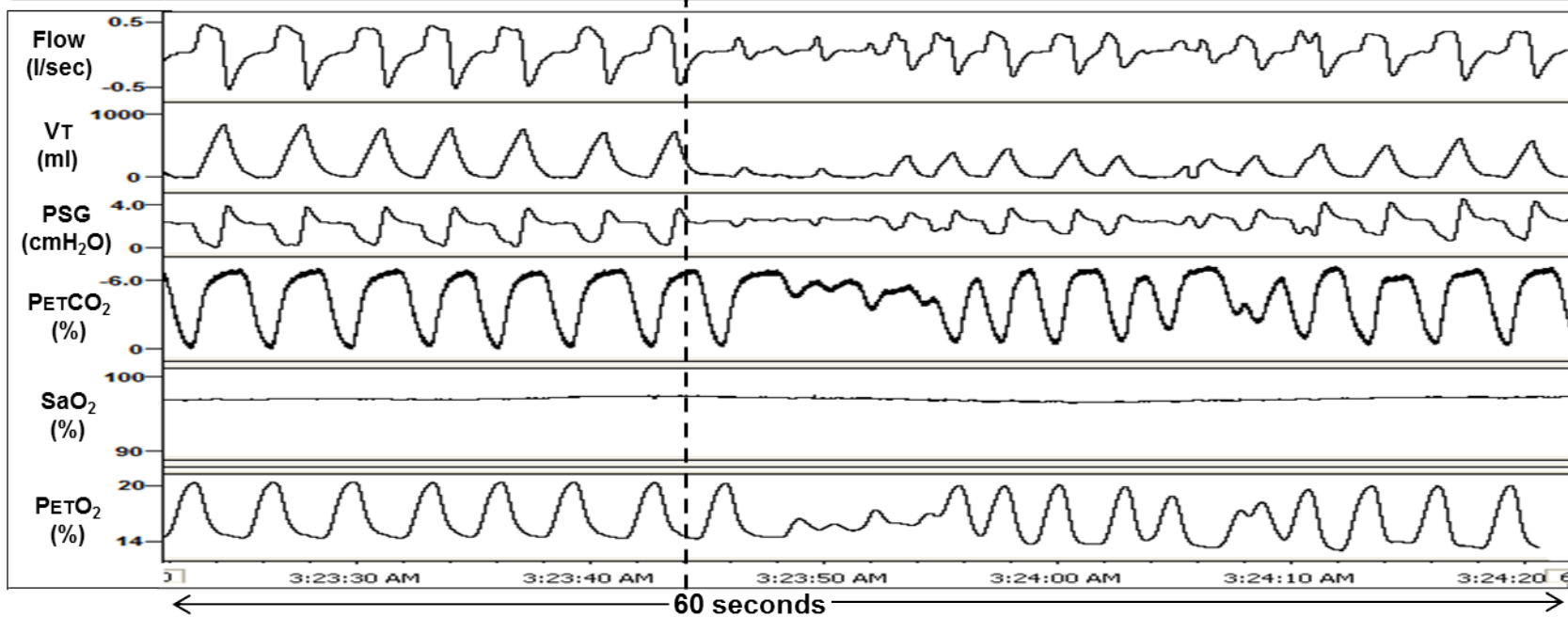

Supplement: Supplementary file 3 [file phy20003-e12490-sd3.pdf]

Theta  $V_T$  % Alpha

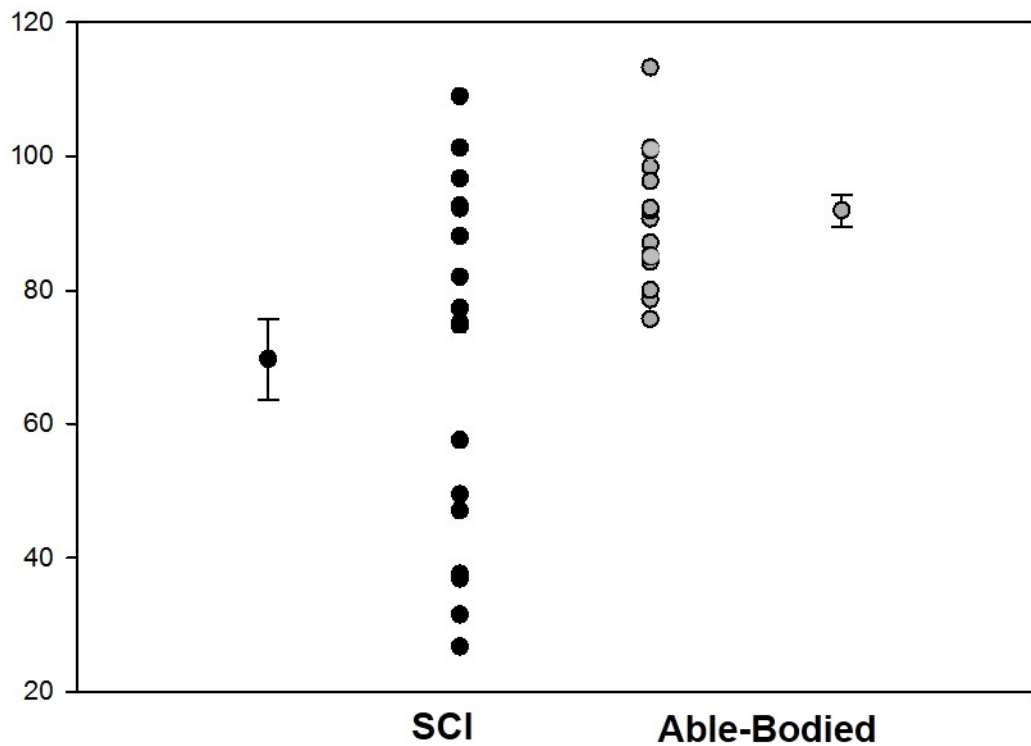

Supplement: Supplementary file 4 [file phy20003-e12490-sd4.pdf]

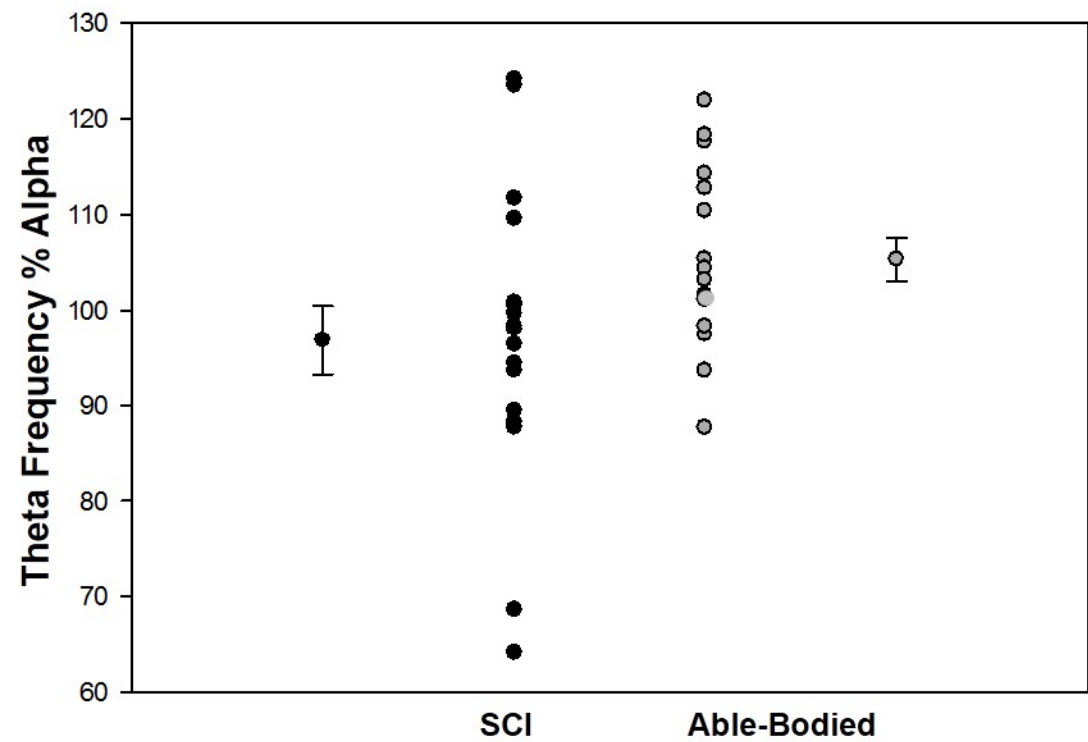

Supplement: Supplementary file 5 [file phy20003-e12490-sd5.pdf]
