# Supplementary material for: Sleep onset hypoventilation in chronic spinal cord injury
Source: Physiol Rep. 2015 Aug 19;3(8):e12490. doi: 10.14814/phy2.12490 (PMC4562576; doi:10.14814/phy2.12490)
Supplement: Supplementary file 6 [file phy20003-e12490-sd6.docx]

| Table 5. Tidal volume during non-REM to REM transitions | | |
| --- | --- | --- |
|  | Non-REM | REM |
| cSCI |  |  |
| 1c | 763.8±93.2 | 710.6±155.4 |
| 2c | 295.5±56.5 | 269.2±106.8 |
| 3c | 232.5±40.9 | 290.4±81.6 |
| tSCI |  |  |
| 1t | 279.9±47.2 | 212.6±128.4 |
| 2t | 249.3±93.6 | 247.3±51.3 |
| Able-Bodied |  |  |
| 1ab | 448.7±42.5 | 322.9±148.0 |
| 2ab | 434.0±22.4 | 394.3±71.1 |
| 3ab | 470.8±26.9 | 153.4±85.3 |
| cSCI: cervical SCI; tSCI: thoracic SCI; Tidal Volume (mL). non-REM: average of the 10 breaths of non-REM sleep just prior to REM onset for each subject ± S.D. REM: average of the first 10 breaths in REM sleep for each subject ± S.D. Each subject is denoted by a number followed by C for cervical SCI, T for thoracic SCI or AB for able-bodied. | | |
